# Small Bowel Obstruction Caused by Entrapment of a Meckel Diverticulum Within an Ileo-Mesenteric Fibrous Band: A Case Report

**DOI:** 10.3390/reports9030286

**Published:** 2026-08-26

**Authors:** Abdallah Shurrab, Awad Dmour, Mouaiad Abu Alika, Muhammad Moosa, Lucian-Cristian Bulat

**Affiliations:** 1 “Grigore T. Popa” University of Medicine and Pharmacy, 700115 Iasi, Romanialucian.bulat@umfiasi.ro (L.-C.B.); 2 Department of General Surgery, “St. Spiridon” County Emergency Clinical Hospital, 1 Independence Boulevard, 700111 Iasi, Romania

**Keywords:** Meckel’s diverticulum, small bowel obstruction, internal hernia, ileo-mesenteric fibrous band, virgin abdomen, case report

## Abstract

**Background and Clinical Significance**: Meckel’s diverticulum is the most common congenital anomaly of the gastrointestinal tract and an uncommon cause of small bowel obstruction in adults, in which the diverticulum or a band arising from it typically constricts the adjacent bowel. Entrapment of the diverticulum itself within a pre-existing fibrous band is exceedingly rare; **Case Presentation**: A 42-year-old man with no previous abdominal surgery presented with colicky abdominal pain, distension and bilious vomiting. Contrast-enhanced computed tomography demonstrated small bowel loops dilated to 35.4 mm with a pelvic transition point of undetermined cause. At emergency laparotomy, a fibrous ileo-mesenteric band delimited a constricting orifice through which the Meckel’s diverticulum had herniated and become strangulated at its base, with secondary obstruction and ischemia of the adjacent ileum. Division of the band restored perfusion; the bowel remained viable and stapled diverticulectomy was performed without segmental resection. The patient recovered without complications. In most reported cases of Meckel’s diverticulum causing small bowel obstruction, the diverticulum or a band arising from it constricts the adjacent bowel. In this case the mechanism is reversed: the diverticulum was the entrapped structure itself, herniating through and strangulating within the orifice of a pre-existing ileo-mesenteric fibrous band. This configuration is exceptionally rare; **Conclusions**: Meckel’s diverticulum should be considered among the possible causes of small bowel obstruction in adults with no history of abdominal surgery. Recognition of this unusual anatomical configuration may facilitate intraoperative identification and appropriate assessment of bowel viability.

## 1. Introduction and Clinical Significance

Meckel’s diverticulum (MD) results from incomplete obliteration of the omphalomesenteric duct and is the most common congenital anomaly of the gastrointestinal tract, with a reported prevalence of 0.3–2.9% [1,2]. It is a true diverticulum arising from the antimesenteric border of the distal ileum, located a mean distance of 52 cm from the ileocaecal valve and measuring a mean of 3 cm in length [2]. The lifetime risk of complications is estimated at 4–6% and decreases with age [3]. Obstruction accounts for approximately 35% of symptomatic presentations in adults, followed by inflammation and hemorrhage [2].

Obstructive complications are conventionally attributed to intussusception with the diverticulum as the lead point, volvulus around a persistent vitelline cord, herniation beneath a mesodiverticular band, axial torsion, diverticulitis with stricture, or incarceration within a groin hernia sac [1,3,4]. In each of these mechanisms the diverticulum, or a band originating from it, constitutes the constricting element. Small bowel obstruction (SBO) in a patient without previous abdominal surgery is uncommon and diagnostically demanding. Because postoperative adhesions, the most common cause of SBO, are absent in these patients [5,6], identification of the underlying cause may be difficult. We report an adult with a virgin abdomen in whom the diverticulum was entrapped and strangulated within the orifice of a pre-existing ileo-mesenteric fibrous band. The report conforms to the SCARE 2023 guideline [7].

## 2. Case Presentation

A 42-year-old Romanian man self-presented to the emergency department of St. Spiridon County Emergency Clinical Hospital, Iași, with acute diffuse colicky abdominal pain, progressive distension, nausea and bilious vomiting. Onset of pain was 2 days before presentation after alcohol consumption. He had no history of previous abdominal surgery. The patient had no history of reccurrent abdominal pain, anemia or gastrointestinal bleeding. The patient had a history of alcohol use disorder, chronic liver disease, a previous episode of acute pancreatitis, and cholelithiasis. Family history was unremarkable. He reported no known allergies and was not receiving regular medication.

On examination, the patient was afebrile and haemodynamically stable but appeared distressed. The abdomen was distended, tympanitic, and diffusely tender, without guarding, rebound tenderness, or a palpable mass. No inguinal, femoral, or umbilical hernia was identified. Based on the clinical presentation, mechanical small bowel obstruction was suspected. Given his history of pancreatitis and gallstone disease, recurrent pancreatitis and biliary pathology were also initially considered.

Laboratory investigations showed a normal leukocyte count of 5.22 × 10^3^/μL, hemoglobin of 16.8 g/dL, C-reactive protein of 3.72 mg/dL, and serum urea of 104 mg/dL, with a normal creatinine concentration of 0.99 mg/dL. The elevated urea concentration, together with the relatively high hemoglobin level, was consistent with dehydration secondary to vomiting and third-space fluid loss.

**Figure 1:**
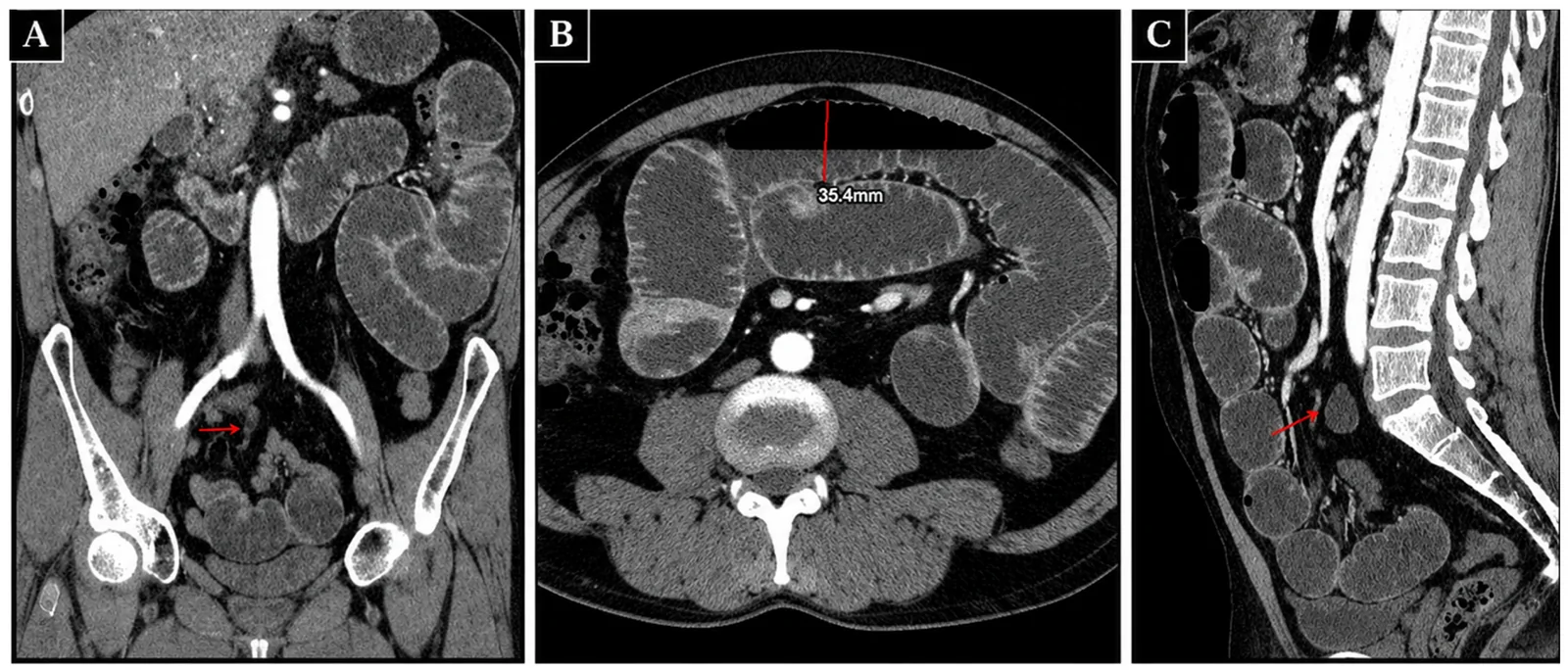
Contrast-enhanced computed tomography findings in small bowel obstruction. (**A**) Coronal contrast-enhanced CT demonstrating multiple dilated small bowel loops, with the arrow indicating the transition point in the right iliac fossa. (**B**) Axial contrast-enhanced CT showing a dilated small bowel loop measuring approximately 35.4 mm, with an air-fluid level. (**C**) Sagittal reformatted contrast-enhanced CT demonstrating the transition point (arrow), with dilated proximal and collapsed distal small bowel loops.

Plain abdominal radiography demonstrated multiple air-fluid levels, predominantly in the left hemiabdomen. Ultrasonography showed fluid-filled small bowel loops measuring up to 49 mm in diameter, with mural thickening of up to 7 mm. Contrast-enhanced abdominopelvic computed tomography confirmed mechanical small bowel obstruction, with fluid-filled small bowel loops measuring up to 35.4 mm, multiple air-fluid levels, and a pelvic transition point followed by collapsed distal bowel (Figure 1). No free intraperitoneal air, pneumatosis, or mass lesion was identified. Because the patient had no history of abdominal surgery, the principal etiological considerations were a congenital or adhesive band, internal hernia, small bowel tumor, inflammatory stricture, or intussusception. The precise cause was not identified preoperatively.

**Figure 2:**
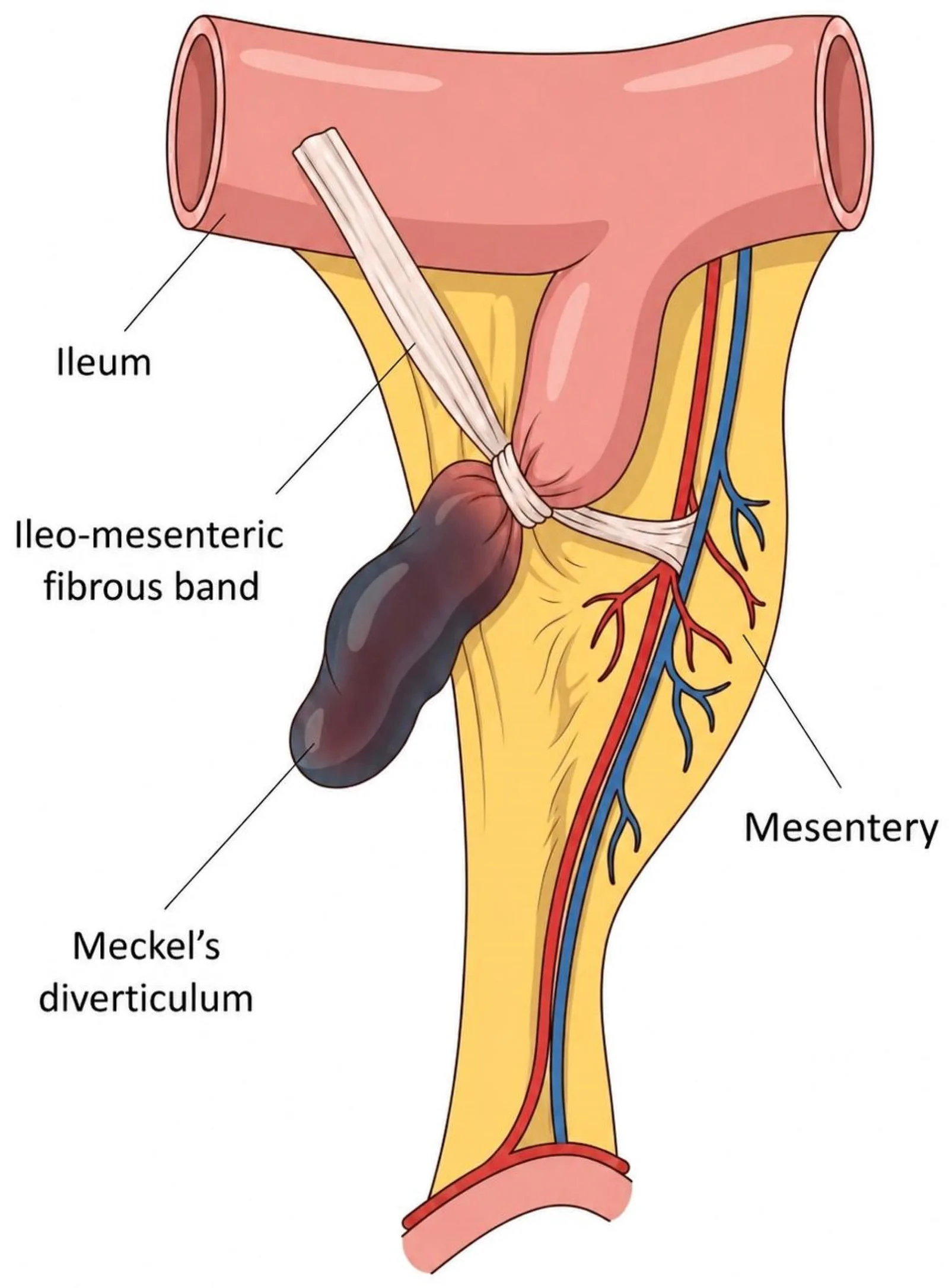
Schematic representation of the proposed anatomical mechanism. An ileo-mesenteric fibrous band extends from the antimesenteric aspect of the ileum to the adjacent mesentery, forming an aperture beneath which the Meckel diverticulum becomes entrapped and constricted near its base, resulting in secondary obstruction of the adjacent ileum. The illustration is schematic and based on the intraoperative findings.

Given the persistence of obstructive symptoms for approximately two days, the presence of a discrete transition point with collapsed distal ileum, and concern for a structural cause of obstruction in a patient without previous abdominal surgery, operative exploration was selected rather than prolonged non-operative management. Following resuscitation, nasogastric decompression, antibiotic prophylaxis and general anesthesia, emergency exploratory laparotomy was performed through a midline incision on the day of admission. Intraoperatively, a Meckel diverticulum was identified approximately 25 cm proximal to the ileocecal valve. A fibrous band extended from the antimesenteric aspect of the ileum proximal to the diverticular base to the adjacent mesentery, forming a bridge-like aperture. The diverticulum had passed beneath this band and was constricted near its base. The adjacent ileal segment was consequently angulated and obstructed and appeared ischemic, although no perforation or necrosis was present. No volvulus, intussusception, or purulent peritoneal fluid was identified. Figure 2 schematically illustrates the proposed anatomical configuration.

**Figure 3:**
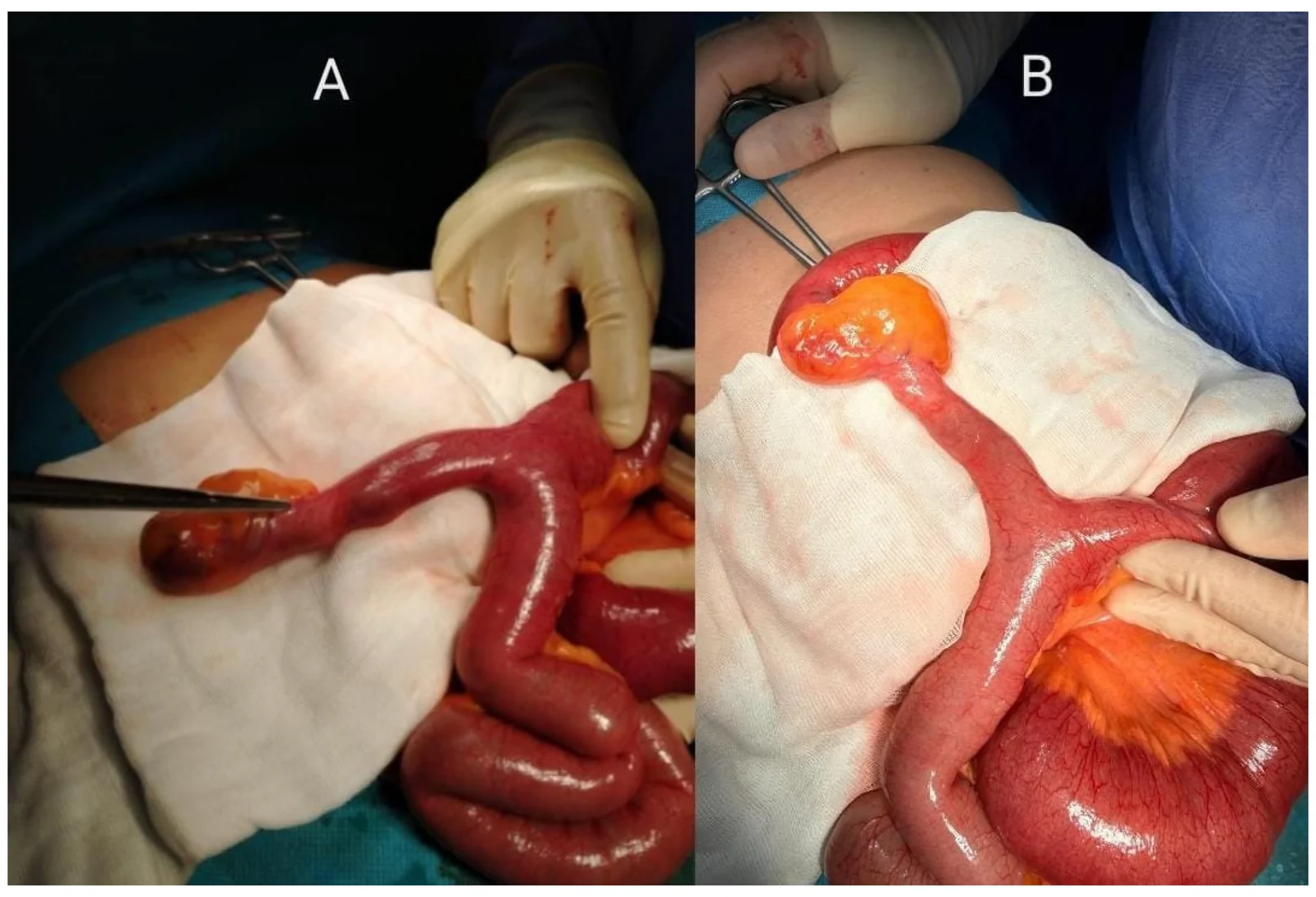
Intraoperative appearance of the Meckel diverticulum and adjacent ileum. (**A**) Intraoperative appearance following release of the constricting fibrous band, demonstrating the Meckel diverticulum and adjacent ileal segment. (**B**) Further intraoperative view demonstrating the elongated Meckel diverticulum and viable adjacent ileum after release of the obstruction.

We performed adhesiolysis, releasing the strangulated diverticulum (Figure 3). After release and warm packing, the ileal segment recovered normal color, peristalsis and mesenteric pulsation and was considered viable; segmental resection was therefore not performed. Diverticulectomy was completed at the base using a linear stapler, and a single drain was placed.

The postoperative course was uneventful, with progressive restoration of intestinal transit and removal of the drain. This is consistent with the low operative morbidity and favorable prognosis reported after resection of Meckel’s diverticulum in large series [8]. No adverse or unanticipated events occurred. The patient was discharged on the seventh postoperative day with a recommendation to follow a healthy diet regimen and avoid any strenuous physical exercise for three months. Histopathological examination confirmed a Meckel diverticulum with areas of gastric metaplasia showing foveolar and oxyntic differentiation. The adjacent adipose tissue demonstrated congestion, recent hemorrhage, liponecrosis, fibrosis, and a mild polymorphous inflammatory infiltrate. No malignancy was identified. Skin staples were removed on day 14 and the patient remained asymptomatic with normal intestinal transit at outpatient review on day 21. The patient was generally satisfied with the treatment plan and all questions and concerns were answered. At the 90-day postoperative follow-up, the patient remained asymptomatic, with no reported recurrent abdominal complaints or obstructive symptoms. Physical examination, laboratory investigations, and ultrasonographic assessment revealed no postoperative complications. The clinical course is summarized in Table 1.

## 3. Discussion

The Mayo Clinic series of 1476 patients identified male sex, age below 50 years, diverticular length exceeding 2 cm and the presence of histologically abnormal tissue as the features most strongly associated with symptomatic presentation [8]. All four were present in this patient, whose 6 cm diverticulum was approximately twice the reported mean length and contained gastric metaplasia. The obstructive mechanism, however, differed from those conventionally described.

In the majority of reported configurations, the diverticulum is the active constricting element. The mesodiverticular band that is the most frequently implicated mechanism in adults, arises from the diverticulum and inserts on the ileal mesentery, leading to the creation of an aperture or a loop through which a bowel loop herniates and strangulates. Histological examination characteristically demonstrates vascular and neural structures within the band, consistent with a vitelline artery remnant [9,10]. In the present case this relationship was inverted; the constricting orifice or loop was formed by a band bridging the antimesenteric wall of the ileum and adjacent mesentery, while the herniated and strangulated structure was the diverticulum itself, with obstruction of the adjacent ileum arising secondarily. A focused literature review identified several anatomically related mechanisms, but the present configuration appears unusual in that the diverticulum itself was entrapped within an ileo-mesenteric fibrous band that was not demonstrably arising from the diverticulum.

The reports most similar to ours, which are compared below and in Table 2, are those of Ben Jabra et al. and Wu et al. [11,12], in which the diverticulum itself was the entrapped structure, yet the anatomical elements and locations were different. Ben Jabra et al. described a diverticulum incarcerated within a Ladd’s band in a patient with an incomplete common mesentery. Wu et al. reported a diverticulum that had herniated through a congenital mesocolic defect; in both cases, a volvulus developed and the bowel became necrotic, requiring segmental resection. Our case is similar in that the diverticulum was the trapped structure, but differs in that it was entrapped by a pre-existing ileo-mesenteric fibrous band, with no intestinal malrotation, Ladd’s band, or mesocolic defect, and no volvulus. An intermediate group is represented by Zaatar et al. and Ebrahimi et al. [13,14], in which a band formed on or tethered the diverticulum and the strangulation occurred at or near the diverticular base; these resemble our case in that the diverticulum lay at the point of constriction, but differ in that the band appeared to arise from the diverticulum rather than being an independent ileo-mesenteric structure. The remaining reports differ more fundamentally because the diverticulum formed part of the constricting apparatus through which a separate bowel loop herniated. Mwila et al. [15] described an ileo-mesenteric band of vitelline origin, whereas Zhang et al. and Pandove et al. [16,17] reported a band or mesenteric structure associated with the diverticulum that created a ring or sac through which adjacent bowel herniated. Henry et al. [18] described an ileo-mesenteric band of vitelline origin and an interloop adhesive band that trapped bowel loops with the diverticulum adjacent. These mechanisms and their distinguishing elements are summarized in Table 2.

The exact origin of the ileo-mesenteric fibrous band could not be established. Histopathological examination was performed on the resected Meckel diverticulum and associated adipose tissue, which demonstrated congestion, recent hemorrhage, liponecrosis, fibrosis, and a mild polymorphous inflammatory infiltrate. However, the divided fibrous band was not submitted or identified separately for histopathological characterization. Consequently, its congenital or acquired origin cannot be determined. The inflammatory and fibrotic changes in the adjacent tissue make an acquired inflammatory mechanism plausible, while a congenital remnant cannot be definitively excluded. Fibrosis following subclinical diverticular inflammation remains a possible explanation [14]. In future similar cases, separate histopathological examination of the band should be considered, as identification of organized vascular and neural elements would support a vitelline origin [9,10].

Computed tomography established the presence and level of obstruction but not its cause, consistent with its recognized limited sensitivity for Meckel’s diverticulum [4,19]. Reported signs favoring the diagnosis include a midline transition point, a blind-ending tubular structure continuous with the distal ileum, a closed-loop configuration and convergence of mesenteric vessels at the transition zone [4,19]. Preoperative diagnosis is achieved in a minority of cases, and exploration remains both diagnostic and therapeutic [3,6]. Stapled diverticulectomy was performed in this patient; segmental resection with anastomosis is generally preferred when the base is broad or ectopic mucosa is suspected because tangential excision may leave heterotopic tissue at the base [3,8,13].

A strength of this report is the complete clinical and operative documentation with intraoperative imaging; its cross-specialty relevance spans emergency surgery and radiology. On the other hand, the principal limitations of this report are the absence of histological characterization of the band in order to detrermine whether its origin was acquired or congenital. Also, as a single observation, it cannot establish the frequency of this mechanism. We recommend further attention to the described mechanism in MD-related SBO patients as well as examining the adhesive band histopathologically in any future similar cases. Given the rarity of such cases, aggregation of individual reports into a pooled analysis would help define its true frequency, associated anatomical variants, and optimal operative strategy.

## 4. Conclusions

Meckel diverticulum should be considered among the possible causes of mechanical small bowel obstruction in adults with no history of abdominal surgery. This case demonstrates an unusual configuration in which the diverticulum itself became entrapped and strangulated within an ileo-mesenteric fibrous band rather than acting as the primary constricting structure. Awareness of such anatomical variants may facilitate intraoperative identification and appropriate assessment of bowel viability. Separate histopathological examination of associated fibrous bands should be considered in similar cases to clarify their congenital or acquired origin.

## Figures and Tables

**Table 1:** Clinical timeline.

Time Point	Clinical Event
Two days before admission	Onset of abdominal pain, distension, nausea, and bilious vomiting
Day of admission	Imaging confirmed mechanical small bowel obstruction with a pelvic transition point
Day of admission	Emergency laparotomy identified a Meckel diverticulum strangulated within an ileo-mesenteric fibrous band
Intraoperative period	Division of the band and stapled diverticulectomy without ileal resection
Postoperative day 7	Discharged without complications
Postoperative day 14	Skin staples removed
Postoperative day 21 Postoperative day 90	Asymptomatic at follow-up; histopathological findings reviewed No complaints and/or complications

**Table 2:** Reported adult and pediatric cases of Meckel’s diverticulum associated with band-related or internal herniation mechanisms of small bowel obstruction, grouped by whether the diverticulum was the constricting or the trapped structure, and compared with the present case.

Report (year)	Age/Sex	Prior Abdominal Surgery	Constricting Structure	Structure Trapped/Herniated	Volvulus	Bowel Outcome
Present case	42/M	None	Pre-existing ileo-mesenteric fibrous band	Meckel’s diverticulum itself	No	Viable; band divided, no resection
Ben Jabra et al. 2023 [11]	54/M	None	Ladd’s band (incomplete common mesentery)	Diverticulum + bowel loop	Yes	30 cm necrosis; resection
Wu et al. 2014 [12]	45/M	None	Congenital transmesocolic defect	Diverticulum(lead point)	Yes	20 cm necrosis; resection
Zaatar et al. 2023 [13]	80/M	None	Adhesive band on the diverticulum	Constriction at diverticular base	No	~5 cm; resection
Ebrahimi et al. 2021, case 2 [14]	56/M	None	Band from diverticular apex to mesentery	Diverticular apex tethered	No	Resection and anastomosis
Mwila et al. 2022 [15]	40/F	None	Congenital ileo-mesenteric band (vitelline)	Bowel loop	No	Viable; band division
Zhang et al. 2026 [16]	6/F	None	Band arising from the diverticulum → ring	Proximal ileum	No	25 cm necrosis; resection
Pandove et al. 2015 [17]	14/M	None	Diverticular mesentery + band → sac	Small bowel loops	No	Resection of adjacent ileum
Henry et al. 2020 [18]	38/M	None	Interloop adhesive band (bowel-to-bowel) + MD	Bowel loops (diverticulum adjacent)	No	10 cm; resection

## Data Availability

The original contributions presented in this study are included in the article. Further inquiries can be directed to the corresponding authors.

## Abbreviations

The following abbreviations are used in this manuscript:

MD	Meckel’s diverticulum
SBO	Small bowel obstruction
CT	Computed tomography
POD	Postoperative day
